# Identification of Urinary Peptide Biomarkers Associated with Rheumatoid Arthritis

**DOI:** 10.1371/journal.pone.0104625

**Published:** 2014-08-21

**Authors:** Angelique Stalmach, Hanna Johnsson, Iain B. McInnes, Holger Husi, Julie Klein, Mohammed Dakna, William Mullen, Harald Mischak, Duncan Porter

**Affiliations:** 1 University of Glasgow, Institute of Cardiovascular and Medical Sciences, Glasgow, United Kingdom; 2 Western General Infirmary, Edinburgh, United Kingdom; 3 University of Glasgow, Institute of Infection, Immunity and Inflammation, Glasgow, United Kingdom; 4 Mosaiques-Diagnostics GmbH, Hannover, Germany; University of Leuven, Rega Institute, Belgium

## Abstract

Early diagnosis and treatment of rheumatoid arthritis are associated with improved outcomes but current diagnostic tools such as rheumatoid factor or anti-citrullinated protein antibodies have shown limited sensitivity. In this pilot study we set out to establish a panel of urinary biomarkers associated with rheumatoid arthritis using capillary electrophoresis coupled to mass spectrometry. We compared the urinary proteome of 33 participants of the Scottish Early Rheumatoid Arthritis inception cohort study with 30 healthy controls and identified 292 potential rheumatoid arthritis-specific peptides. Amongst them, 39 were used to create a classifier model using support vector machine algorithms. Specific peptidic fragments were differentially excreted between groups; fragments of protein S100-A9 and gelsolin were less abundant in rheumatoid arthritis while fragments of uromodulin, complement C3 and fibrinogen were all increasingly excreted. The model generated was subsequently tested in an independent test-set of 31 samples. The classifier demonstrated a sensitivity of 88% and a specificity of 93% in diagnosing the condition, with an area under the receiver operating characteristic curve of 0.93 (p<0.0001). These preliminary results suggest that urinary biomarkers could be useful in the early diagnosis of rheumatoid arthritis. Further studies are currently being undertaken in larger cohorts of patients with rheumatoid arthritis and other athridities to assess the potential of the urinary peptide based classifier in the early detection of rheumatoid arthritis.

## Introduction

Rheumatoid arthritis (RA) is a systemic autoimmune condition that primarily affects the joints and can lead to joint damage, disability and premature mortality. Early diagnosis and treatment of RA are associated with better patient response to treatment [1], [2], reduced co-morbidity and lower mortality [3]. In the initial stages of the disease, accurate diagnosis can be challenging. In 2010, the American College of Rheumatology and European League Against Rheumatism (ACR/EULAR) developed a new approach to classifying RA based on scoring criteria [4]. This classification system improves sensitivity for the early detection of the disease compared to the former ACR 1987 classification criteria. However it has been shown to lead to both over- and under-diagnosis [5]. The biomarkers rheumatoid factor (RF) and anti-citrullinated protein antibodies (ACPA) form one of the current classification criteria, but in isolation the markers lack sensitivity [6]. Novel biomarkers which could assist in accurate, early diagnosis would facilitate more effective early intervention whilst limiting exposure to disease modifying therapy in patients otherwise destined to remit spontaneously. Recent studies have sought novel RA biomarkers in peripheral blood and synovial fluid [7]–[10]. Despite early promises, none of these approaches have yet yielded combinations of biomarkers with better specificity and sensitivity than ACPA used alone.

A novel approach for identification of diagnostic biomarkers in RA uses capillary electrophoresis coupled to mass spectrometry (CE-MS). This method has already enabled the identification of urinary biomarker classifiers for the diagnosis of diseases like chronic kidney disease [11], acute kidney injury [12], stroke [13], and cardiovascular diseases [14]. It allows classification of case versus control groups with good accuracy [15]. The use of urine rather than blood for the identification of biomarkers has several advantages, including non-invasive sample collection, a high stability due to absence of proteolytic agents and a low dynamic range of analyte concentration which facilitates the detection and quantification of peptides [16]. Furthermore, discovery of proteomic biomarkers may be useful in understanding the molecular mechanisms involved in the onset and progression of disease [17]. In this study, we aimed to identify potential biomarkers for the early diagnosis of RA. We hypothesized that RA-specific peptides would be measured in urine samples of patients and produce a unique fingerprint of peptides compared to healthy controls. This was achieved by comparing the urinary peptide profile of patients with RA with that of healthy controls. Our secondary objective was to identify the proteases involved in generating the RA-specific peptide fragments, using an in silico approach.

## Materials and Methods

### RA population and data collection

Patients are recruited to the Scottish Early RA (SERA) inception cohort if they have newly diagnosed undifferentiated arthritis or rheumatoid arthritis. Detailed demographic and clinical data are recorded; samples of blood, urine and synovial fluid are taken every 6 months, and stored for future analyses.

Patients enrolled in SERA who fulfilled the ACR/EULAR diagnostic criteria for RA at diagnosis were identified, and 25 ACPA positive (>20 units) and 24 ACPA negative (<7 units) patients were selected at random. Clinical information on gender, age, disease duration from onset of symptoms, 28 joint count disease activity score (DAS28) [18], health assessment questionnaire (HAQ), C-reactive protein (CRP), ACPA and RF values were collected. Laboratory tests had been analysed in routine National Health Service (NHS) laboratories across Scotland.

### Control population

Controls were volunteers who agreed to participate in nutritional-based interventions, were at least 18 years of age, non-smokers and in general good health. They were enrolled in other on-going proteomic studies and had given written informed consent prior to starting the studies.

### Ethics statement

Cases provided enduring and generic written consent for their samples to be used in analyses, and the SERA study was approved by the West of Scotland Research Ethics Committee. Controls gave written informed consent prior to participating in on-going proteomic studies approved by the University of Glasgow Faculty of Medicine Ethics Committee.

### Urine collection

For both cases and controls, spot urine samples were collected in sterile containers at the time of the study visit. One millilitre aliquots of unprocessed samples were stored at −80°C prior to being processed as recommended by the European Kidney and Urine Proteomics and Human Kidney and Urine Proteome Project and described previously [16]. Most samples were frozen within 4 hours of collection but samples from remote parts of Scotland were couriered in chilled containers overnight. Once frozen, samples were left to defrost at room temperature, occasionally mixing by gentle inversion, and samples were defrosted only once.

### Urinary proteomic analysis

The urine samples were prepared as previously described by removing large proteins (>20 kDa), urea, electrolytes and salts, and by enriching polypeptides [11]. Briefly, 700 µL of urine were defrosted with the addition of 0.1% PMSF saturated in ethanol and diluted with 700 µL of a solution containing 2 M urea, 0.1 M NaCl, 10 mM NH_4_OH and 0.02% SDS. The mixture was then filtered through a 20 kDa MW cut-off ultra-centrifugation filter device (Sartorius Stedim UK Ltd, United Kingdom) at 2,600 × *g* for one hour at 4°C. A volume of 1.1 mL of the filtrate was then loaded onto a pre-equilibrated PD-10 desalting column (GE Healthcare, Sweden) and eluted using 0.01% aqueous NH_4_OH. The eluate was subsequently freeze-dried and stored at 4°C prior to being resuspended in HPLC-grade water to a final protein concentration of 2 mg/mL for capillary electrophoresis-mass spectrometry analysis.

### Capillary Electrophoresis-Mass Spectrometry (CE-MS) analysis and Data processing

CE-MS analysis was performed as previously described using a P/ACE MDQ capillary electrophoresis system (Beckman Coulter, Fullerton, USA) on line coupled to a MicroTOF MS (BrukerDaltonic, Bremen, Germany) [19]. Samples were injected hydrodynamically at 2.0 psi for 99 sec (ca. 250 nL) and separation of peptides was achieved by reverse polarity at 25 kV for the first 30 min, and with increasing pressure (up to 0.5 psi) for another 34 min. The cartridge temperature was maintained at 25°C. Running buffer contained 79∶20∶1 (v/v) deionised filtered (0.2 µm) water, acetonitrile and formic acid. Sheath liquid consisted of 30% 2-propanol and 0.4% formic acid in deionised filtered (0.2 µm) water. The ESI sprayer (Agilent Technologies, Palo Alto, CA, USA) was grounded, and the ion spray interface potential was set between −4 and −4.5 kV. Spectra were accumulated every 3 seconds over a range of mass-to-charge ratios from 50 to 3000. Details on accuracy, precision, selectivity, sensitivity, reproducibility, and stability of the CE-MS method have been previously described [11], [20]. MosaiquesVisu was used to analyse the CE-MS data [21]. Peptides are initially characterised by their molecular mass, CE-migration time, and ion signal intensity (amplitude) value. Internal standard peptides were used for calibration, as previously described [22]. All detected peptides were deposited, matched, and annotated in a MicrosoftSQL database, allowing for further analysis and comparison between case and control groups.

### Statistical analysis and classifier development

Of the initial cohort of RA patients (n = 49) and healthy controls (n = 45), 33 cases and 30 controls were selected at random to establish a panel of RA-specific urinary peptides. After testing for normal distribution, continuous data were compared by the Mann-Whitney test, as this test has proven to be of superior statistical power in proteomics datasets [23]. This test is particularly suited for proteomics data as such data suffer from missing values leading to non-normal skewed distributions even after log-transformation. A p-value of <0.05 was considered to be statistically significant, after correction for multiple testing. Only peptides with a frequency of at least 40% in either group were considered for further analysis. In order to control for the false discovery rate at 0.05, the p-values were adjusted by the Benjamini and Hochberg method [24] implemented in the Bioconductor package multtest [25].

Correlative association between significantly excreted peptides and potential confounding factors such as age and gender was assessed in both case and control groups using Spearman’s rank coefficient correlation (age) and Mann-Whitney test (gender) followed by p-value adjustment using the Benjamini and Hochberg method as previously described. Due to the small sample size of the study, we further analyzed the correlation of the potential biomarkers with age and gender in a cohort of 500 healthy patients from our database.

Significantly differentially excreted peptides were subsequently identified using MS-MS and used to develop a biomarker model to classify between RA and non-RA patients. The remaining 31 samples (16 cases and 15 controls) were used as an independent validation subset in the support-vector-machine (SVM) based MosaCluster software [14], [19]. The sensitivity, specificity and area under the receiver operating characteristic (ROC) curve of the resulting model were calculated using MedCalc version 12.1.3.0 (MedCalc Software bvba, Belgium).

The SVM classifier uses the log transformed intensities of n peptides as coordinates in an n dimensional space. It then builds an n-1 hyper plane that spans this space by performing a quadratic programming optimization of a Lagrangian using the training labels only while allowing for samples to lie on the wrong side of the plane. For such misclassification, the SVM introduces a cost parameter C. Due to the fact that non separable problems in low dimensions may be separable in higher dimensions, the SVM uses the so-called Kernel-trick to transform the samples to a higher dimensional space. Mosacluster uses the standard radial basis functions as kernel. These functions are Gaussians with the parameter gamma controlling for the width. The optimal parameters C and gamma are found via a leave-one-out cross validation error estimation. Implantation of SMV is popular in data mining software, and the Kernel-based Machine Learning Lab (kernlab) package in R in particular is used as a versatile tool for building SVM-based classifiers [26].

### Proteases prediction

In order to link urinary fragments to the proteases involved in their generation, a predictive analysis was carried out using Proteasix. This is an open-source tool used to predict the proteases involved in naturally occurring peptide generation in silico, as previously described [27]. Briefly, Proteasix is a cleavage site database that can associate proteases with their corresponding cleavage site sequences based on octopeptides (P4P3P2P1-P1′P2′P3′P4′). Each peptide is described by the respective substrate SWISS-PROT identifier or name, peptide start and peptide end. The search predicts protease association with 0 and up to 3 mismatches in the cleavage site sequence. After entering the peptide list, the tool aligns each peptide sequence with the full-length SWISS-PROT sequence to identify N- and C-term cleavage sites. Each cleavage site is searched in the database to retrieve all predicted protease/cleavage site combinations. This required the generation of a discovery matrix of unique peptide versus protease, and every matrix point is either 0 (no cut), 1 (cut) or 2 (cuts both N-and C-terminus). The matrix was subsequently divided between peptides that were up- or down-regulated based on the absolute fold-change values, and the occurrences were computed by summing the matrix points. Data were assessed using a mathematical approach of frequency analysis. Frequency analysis uses the calculated frequencies of protease association with peptides per protease in either up- or down-regulated groups. The analysis was done using the normal/equal distribution as a reference. Frequency scores were calculated, per protease, using the absolute ratios of the difference over the sum of the frequencies of peptide occurrences that were found up- and down- regulated, multiplied by the difference of occurrences in the up- and down-regulated groups. This mathematical model provides an indication of the distribution of peptide occurrences from the normal distribution, where clustering of peptides around the normal distribution line being interpreted as a lack of specificity in the protease activity.

## Results

### Descriptive data

Median and interquartile ranges are shown in Table 1 for age, duration of symptoms, ACPA, RF, DAS28, HAQ and CRP values for the training and test sets. The patients all fulfilled the 2010 ACR/EULAR diagnostic criteria for RA [4], and had a median duration of symptoms of 113 days in the training set and 91 days in the test set. Most patients had moderate to severe disease activity in both groups, with a DAS28 of greater than 3.2 [28]. In the training set, 48% were positive for ACPA compared to 56% in the test set. A preliminary analysis investigating the correlation between DAS28, HAQ and CRP revealed a poor correlation between CRP and DAS28, and CRP and HAQ (coefficient of determination between DAS28 and HAQ of 0.310 with p-value = 0.001; coefficient of determination between CRP and DAS28 of 0.053 with p-value = 0.124; coefficient of determination between CRP and HAQ of 0.126 with p-value = 0.015).

**Table 1:** Baseline characteristics of the training and test set populations (case and control)^1^.

	RA cohort		Controls	
	Training set (n = 33)	Test set (n = 16)	Training set (n = 30)	Test set (n = 15)
Age (Years)^2^	59 (39; 65)	58 (55; 68)	31 (23; 60)	36 (29; 58)
Female^3^	23 (70%)	13 (76%)	11 (37%)	10 (67%)
Duration of disease (Days)	113 (74; 261)	91 (54; 225)	nd	nd
ACPA positive	16 (48%)	9 (56%)	nd	nd
ACPA (Units)	5.7 (3; 161)	47 (2; 214)	nd	nd
RF^4^ positive	11 (73%)	6 (86%)	nd	nd
RF^4^ (Units)	15 (11; 30)	20 (18; 55)	nd	nd
DAS28 score	5.5 (4.3; 6.2)	4.5 (3.9; 5.5)	nd	nd
HAQ score	1.1 (0.8; 1.9)	1.1 (0.5; 1.6)	nd	nd
CRP abnormal	19 (63%)	11 (73%)	nd	nd
CRP (mg/l)	20.5 (6. 0; 41.0)	19 (8.3; 38.5)	nd	nd

nd, not determined; ACPA, anti-citrullinated protein antibodies; RF, rheumatoid factor; DAS28, 28 joint count disease activity score; HAQ, health assessment questionnaire score; CRP, C-reactive protein.

1 Differences between training set and test set within both RA and control groups were not statistically significant (Mann-Whitney for continuous values and Chi Square for categorical values; p<0.05) with the exception for the proportion of female in the control group between the training and test sets (p<0.05).

2 Difference in the median age value between groups is statistically significant between RA and control groups of the training set (p = 0.0023) and between RA and control groups of the test set (p = 0.0059).

3 Difference in the gender distribution between groups is statistically significant between RA and control groups of the training set (p<0.01) but not between RA and control groups of the test set (p>0.05) (Chi Square test).

4 Data missing for 18 patients in the training set and 9 patients in test set, percentage refers to proportion of patients tested.

### Urinary biomarkers associated with RA

**Figure 1:**
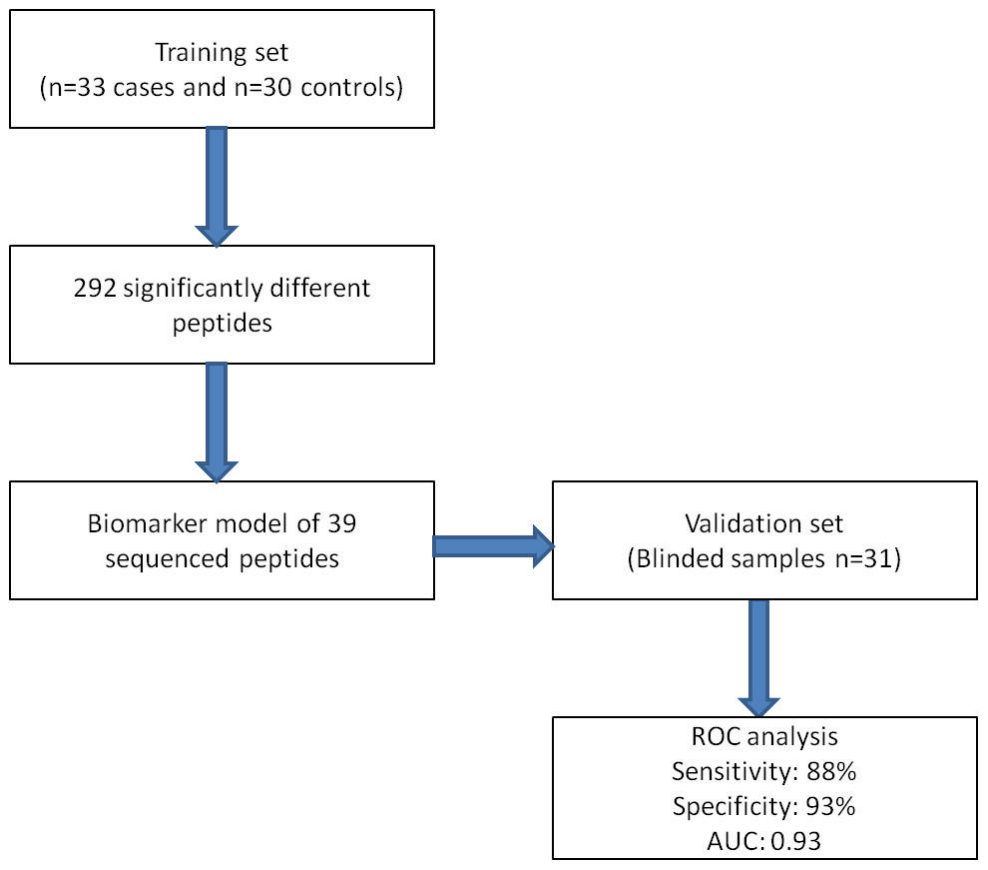
Work flow used for the determination of urinary biomarkers associated with RA.

The work flow used for establishing urinary biomarkers associated with RA is shown in Figure 1. In order to establish potential urinary biomarkers associated with RA, urines samples from 33 RA patients and 30 healthy volunteers were run using CE-MS and analysed for their peptidomic profile.

A total of 292 peptides were significantly differentially regulated in the RA patients compared to controls, after adjustment for multiple testing using the Benjamini-Hochberg procedure for false discovery rate. Of the 292 potential biomarkers, 70 peptides were identified by MS/MS sequencing.

Of these, 34 were found in lower (Table 2) and 36 in higher concentrations (Table 3) in RA patients. Peptide fragments found in significantly lower abundance were identified as protein S100-A9, gelsolin, alpha-1-microglobulin, C-X-C motif chemokine 16, mucin-1 subunit alpha, carboxypeptidase A1 and T calcium channel alpha 1G subunit variant 249. Peptide fragments with higher abundance in RA urine were myosin light chain 3, uromodulin, vesicular integral-membrane protein VIP36, complement 3, fibrinogen alpha chain, clusterin and CD99 antigen. There were also proteins from which peptide fragments were found in both higher and lower concentrations in patients with RA and these were fragments of collagen 1A1 and 1A3, sodium/potassium transporting-ATPase subunit gamma and haemoglobin subunits.

**Table 2:** Urinary peptides which were significantly less abundant in patients with RA.

Fold change	Sequence	Identification
0.018	PpGpPGKNGDDGEAGKPG	Collagen alpha-1(I) chain
0.047	SpGERGETGPpGPA	Collagen alpha-1(III) chain
0.074	VADEAQVQKVKELEDLEHLQ	Carboxypeptidase A1
0.117 *	PpGKNGDDGEAGKPGRpGERGppGP	Collagen alpha-1(I) chain
0.138	pGLPGKAGASGFPGTKGEMGmmGPPGPpGP	Collagen alpha-5(IV) chain
0.138	HAHKLRVDPVNF	Hemoglobin subunit alpha
0.151	GEAGKpGEQGVpGDLGApGP	Collagen alpha-1(I) chain
0.151	TGLSmDGGGSPKGDVDP	Sodium/potassium-transporting ATPase subunit gamma
0.176	VVHTNYDEY	Alpha-1-microglobulin
0.183	EAGENQKQPEKNAGPTAR	C-X-C motif chemokine 16
0.270 *	TTLASHSTK	Mucin-1 subunit alpha
0.312	NpGPPGpSGSpGKDGPpGPAG	Collagen alpha-1(III) chain
0.383	EDLDTNADKQLSFEEF	Protein S100-A9
0.399 *	NRGERGSEGSPGHpGQPGPpGPPGApGP	Collagen alpha-1(III) chain
0.421 *	PpGKNGDDGEAGKPGRpGERGppGPQ	Collagen alpha-1(I) chain
0.432 *	EGSpGRDGSpGAKGDRG	Collagen alpha-1(I) chain
0.439	GSpGSpGPDGKTGPpGPAG	Collagen alpha-1(I) chain
0.456	LSSHIANVERVPFDAATLHTSTA	Gelsolin
0.460 *	DQGPVGRTGEVGAVGpPGFAGEKGPSGEAGTAGPpGTpGP	Collagen alpha-2(I) chain
0.460 *	GLpGTGGpPGENGKpGEPGPKG	Collagen alpha-1(III) chain
0.466	SDGLAHLDNLKG	Hemoglobin subunit delta
0.504	DGVPGKDGPRGP	Collagen alpha-1(III) chain
0.512 *	SpGSPGPDGKTGpP	Collagen alpha-1(I) chain
0.514 *	DGPpGRDGQpGHKG	Collagen alpha-2(I) chain
0.540	ApGPAGSRGApGPQGpRGDKGETGERG	Collagen alpha-1(III) chain
0.548	DpGKNGDKG	Collagen alpha-2(I) chain
0.579 *	pPGADGQPGAKGEpGDAGAKGDAGPpGPAGPAGPPGPIG	Collagen alpha-1(I) chain
0.580 *	GEHNPFKGAI	T calcium channel alpha 1G subunit variant 249
0.617	DDGEAGKpGRpG	Collagen alpha-1(I) chain
0.623 *	GKNGDDGEAGKPGRpGERGPpGp	Collagen alpha-1(I) chain
0.632 *	SpGSPGPDGKTGPpGPAG	Collagen alpha-1(I) chain
0.667 *	PpGPPGPpGPPGPPS	Collagen alpha-1(I) chain
0.700 *	pPGADGQpGAKGEPGDAGAKGDAGPpGPAGPAGPpGPIG	Collagen alpha-1(I) chain
0.733 *	pPGEAGKpGEQGVPGDLG	Collagen alpha-1(I) chain

*Peptides not included in the RA classifying biomarker model.

**Table 3:** Urinary peptides which were found in significantly higher concentration in patients with RA.

Fold change	Sequence	Identification
1.398 *	DGQpGAKGEpGDAGAKGDAGPpGP	Collagen alpha-1(I) chain
1.452 *	EpGSpGENGApGQmGPR	Collagen alpha-1(I) chain
1.522	NSGEpGApGSKGDTGAKGEpGpVG	Collagen alpha-1(I) chain
1.613 *	SGHPGSPGSPGYQGPpGEPGQAGPSGPpGP	Collagen alpha-1(III) chain
1.705	ApGGKGDAGApGERGPpG	Collagen alpha-1(III) chain
1.732 *	NGEpGGKGERGApGEKGEGGPpG	Collagen alpha-1(III) chain
1.749 *	PAPAPPPEPERPKEVE	Myosin light chain 3
1.816	AGERGHPGAPGpSGSpGLPGVPGSMGDMVNYDEIK	Collagen alpha-1(XVI) chain
1.857 *	KGDRGETGpAGPPGApGAPGAPGPVGP	Collagen alpha-1(I) chain
1.960	NGApGEAGRDGNpGNDGPpG	Collagen alpha-2(I) chain
1.984	PpGDEGEmAIISQKGTpGEpGP	Collagen alpha-4(IV) chain
2.074 *	ADGQpGAKGEpGDAGAKGDAGppGP	Collagen alpha-1(I) chain
2.142	SGSVIDQSRVLNLGPITRK	Uromodulin
2.421	QGKTGpPGPPGVVGpQGPTGETGPMGERGHpGPpGP	Collagen alpha-1(V) chain
2.426	NGEpGGKGERGApGEKGEGGppG	Collagen alpha-1(III) chain
2.434 *	GPpGEAGKpGEQGVP	Collagen alpha-1(I) chain
2.607 *	GPpGKNGDDGEAGKPG	Collagen alpha-1(I) chain
2.942	TPEEKSAVTALWGKVNVDEV	Hemoglobin subunit beta
3.084	IDQSRVLNLGPITRK	Uromodulin
3.242 *	ADGQpGAKGEpGDAGAKGDAGPpGPAGP	Collagen alpha-1(I) chain
3.681 *	SGEpGApGSKGDTGAKGEpGP	Collagen alpha-1(I) chain
3.699	GEVGpAGSpGSNGApGQRGEPGPQGHAGAQGPPGpPG	Collagen alpha-1(III) chain
3.910 *	GppGPpGPAGKEG	Collagen alpha-1(I) chain
3.928	VIDQSRVLNLGPIT	Uromodulin
4.016	SGSVIDQSRVL	Uromodulin
4.080	NSGEpGApGSKGDTG	Collagen alpha-1(I) chain
4.547 *	GPpGPTGPGGDKGDTGPpGP	Collagen alpha-1(III) chain
5.569 *	LSMDGGGSPKGDVDP	Sodium/potassium-transporting ATPase subunit gamma
7.564 *	GDpGPpGPpGPpG	Collagen alpha-1(XV) chain
8.523	pGPQGPLGKPGAPGEPGPQG	Collagen alpha-1(VIII) chain
8.928	FGASAGTGDLSDNHDIISMK	Vesicular integral-membrane protein VIP36
11.494	EGVQKEDIPPADLSDQVPDTESETRILLQGTPVA	Complement C3
15.096	RPGApGPAGARGNDGATGAAGPPGPTGpAGpP	Collagen alpha-1(I) chain
16.970	DEAGSEADHEGTHSTKRGHAKS	Fibrinogen alpha chain
23.336	FDSDPITVTVPVEV	Clusterin
27.407	NPPKPMPNPNPNHPSSSGS	CD99 antigen

*Peptides not included in the RA classifying biomarker model.

### Model of RA-specific classifier biomarkers

As the RA population and the controls showed systematic differences in age and gender, their confounding aspect was checked by correlating each of the 70 biomarkers with age and gender in a cohort of 500 healthy patients from our database. This resulted in 31 biomarkers showing a correlation with age, gender or both (see Table S1). These biomarkers were therefore excluded from the RA classifier biomarker model. The biomarker model was established using the remaining 39 sequenced peptides that were significantly different between controls and cases. Accuracy of the model in the training set was 100% when tested employing complete take-one-out crossvalidation.

**Figure 2:**
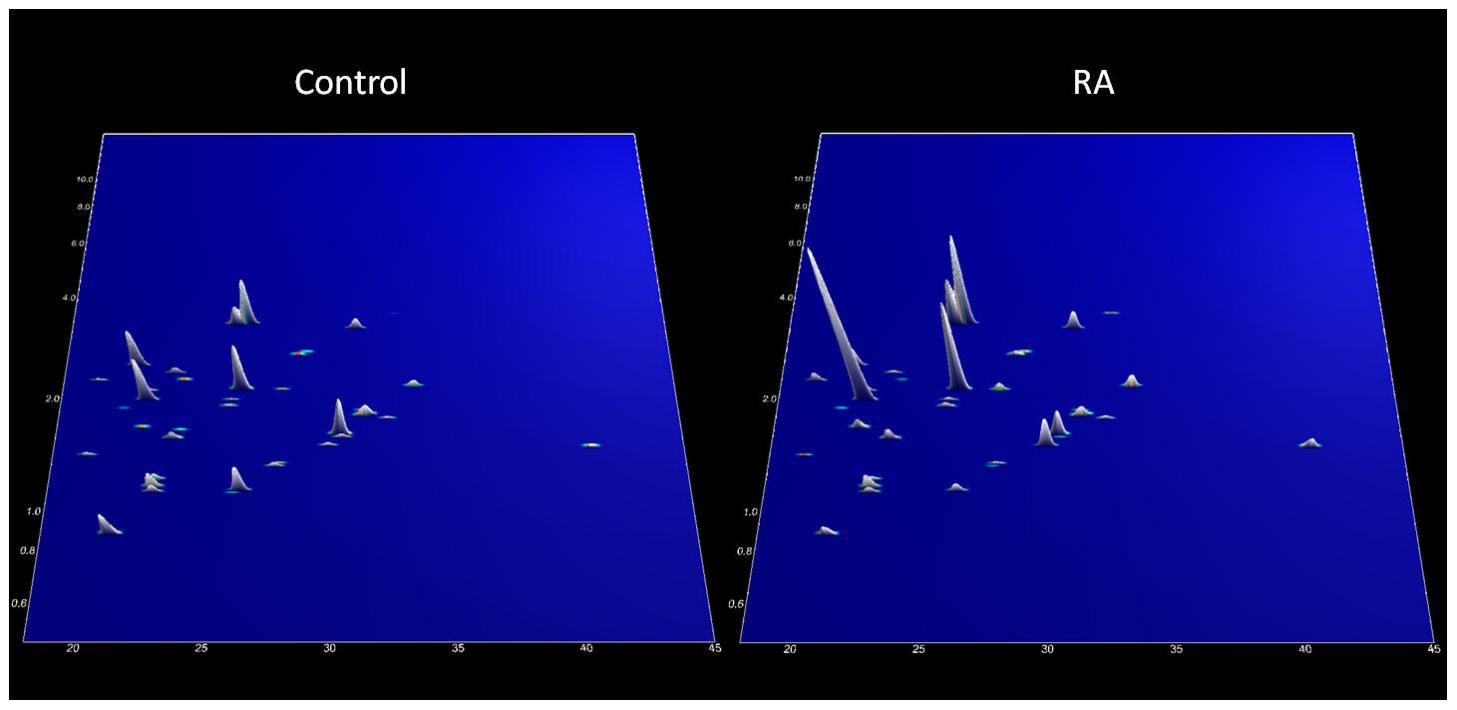
Urinary polypeptide signatures in cases and controls from the validation set based on 39 significantly different sequenced peptides. Normalized molecular weight (500–15000 Da) in logarithmic scale is plotted against normalized migration time (18–45 minutes). The mean signal intensity of the polypeptide peak is given in 3-dimensional depiction (n = 15 controls and 16 cases).

When tested on the blinded test set of 31 samples (16 cases and 15 controls), the proteomic signal was significantly different between groups, and the polypeptidic profiles obtained are shown in Figure 2. Accuracy of the model in the independent test set was 91%, and an area under the curve (AUC) of 0.93 on ROC analysis. Median value of the 39 biomarker classifier model was 0.955 (−2.563; 2.394) with sensitivity of 88% and specificity of 93% for identification of RA (p<0.0001). Median values (range) of the classifier model obtained for the case and control groups were 1.542 (0.175; 2.394) and −0.087 (−2.563; 1.809) respectively.

### Prediction of changes in protease activity based on RA-associated urinary biomarkers

**Figure 3:**
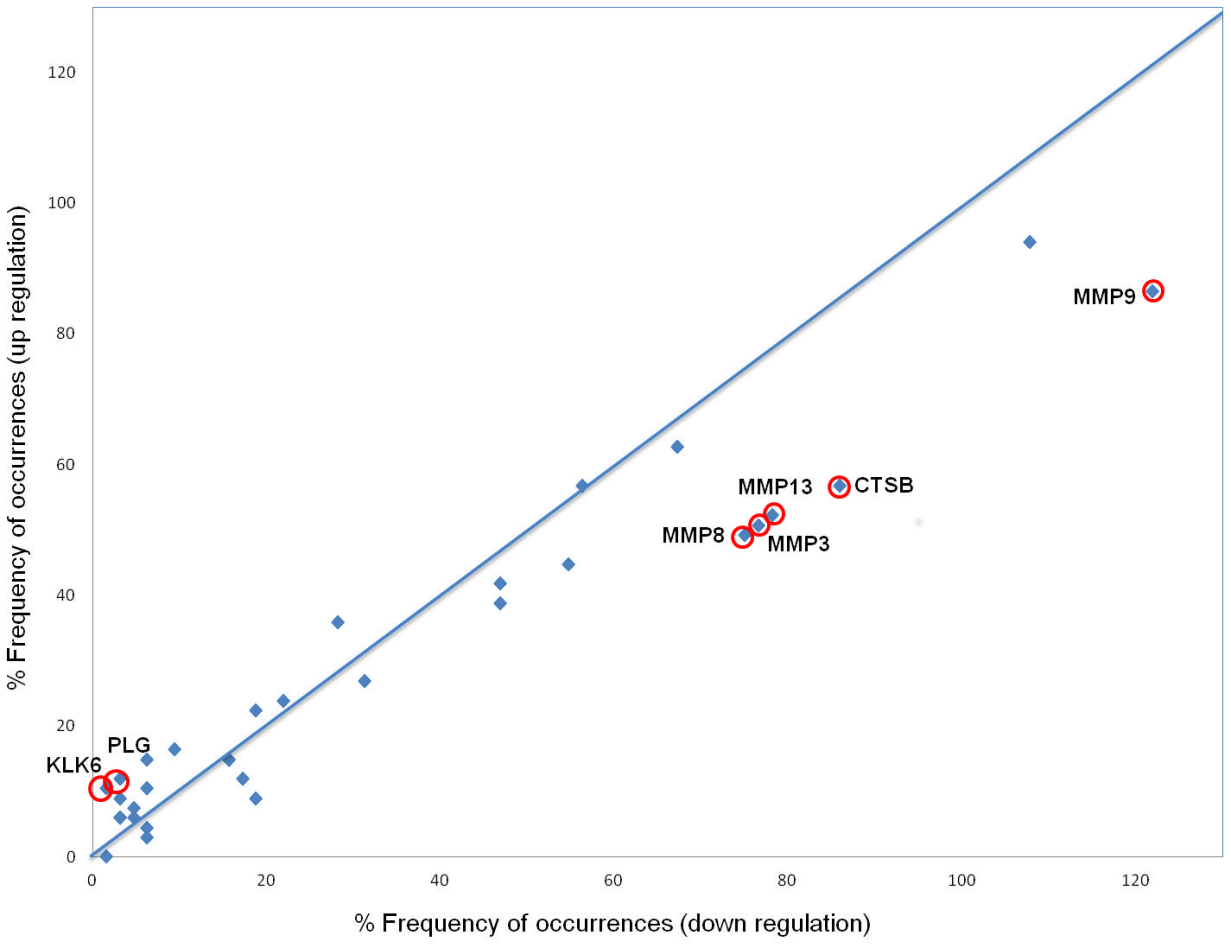
Graphical representation of the frequency distribution of proteases with modified activity associated with RA. Percentage frequency of peptide occurrences in the down-regulation group is plotted on the x-axis, whereas the percentage frequency of occurrences in the up-regulated group is plotted on the y-axis. Circled data points represent the proteases which activity is the most affected in RA compared to that of healthy controls (see Table 4).

Predicted changes in protease activity, based on the association between a protease and its corresponding cleavage site sequence in the octapeptide form, was carried out on peptides which were significantly differentially regulated on the entire cohort of cases (n = 49) and controls (n = 45). Proteasix search and subsequent analysis resulted in the identification of 131 peptides associated with 32 proteases, resulting in 1271 protease-peptide pairs. The frequency distribution analysis presented here (computed as frequency scores) provides a mathematical modelling of the data taking into account the frequency of peptide occurrences per protease weighed by the difference of occurrences. This analysis, as opposed to a statistical approach based on chi-squared frequency distribution or test of independency, takes into account not only the frequency of distribution but also the difference of occurrences, therefore discarding proteases resulting in a similar number of peptides found equally up- and down- regulated. As plotted in Figure 3, proteases found the furthest away from the normal distribution line (demonstrating an increased specificity in cleavage activity) and with a greater difference in the number of occurrences between the up- and down- regulated groups (as indicated by the higher frequency score in Table 4, with negative scores emphasising a down-regulation) are potential candidates involved in the pathophysiology of RA. Amongst the 32 proteases associated with urinary fragments of RA-related peptides, the potential predicted activities of kallikrein 6 (KLK6) and plasminogen (PLG) were up-regulated, whereas the associated activities of matrix metalloproteinase 3 (MMP3), MMP8, MMP9 and MMP13 and cathepsin B were down-regulated in cases compared to controls (Table 4).

**Table 4:** Predictive analysis of changes in protease activity associated with peptides differentially regulated in RA 1.

Protease	occ(up)[N(up) = 67]	occ(down)[N(down) = 64]	% frequency(up)	% frequency(down)	% frequencydifference ratio	Frequencyscores
**Kallikrein 6**	**7**	**1**	**10.5**	**1.6**	**74.0**	**443.9**
**Plasminogen**	**8**	**2**	**11.9**	**3.1**	**58.5**	**351.1**
**Cathepsin B**	**38**	**55**	**56.7**	**85.9**	**20.5**	**−348.2**
**MMP9**	**58**	**78**	**86.6**	**121.9**	**16.9**	**−338.8**
**MMP8**	**33**	**48**	**49.3**	**75.0**	**20.7**	**−310.8**
**MMP13**	**34**	**49**	**50.8**	**76.6**	**20.3**	**−304.2**
**MMP3**	**35**	**50**	**52.2**	**78.1**	**19.9**	**−297.9**
Prolyl endopeptidase	10	4	14.9	6.3	41.0	245.8
ADAMTS4	6	12	9.0	18.8	35.4	**−**212.1
Kallikrein 4	6	2	9.0	3.1	48.3	193.1
Granzyme A	11	6	16.4	9.4	27.3	136.5
KLK3	0	1	0.0	1.6	100.0	**−**100.0
MMP20	7	4	10.5	6.3	25.1	75.4
Cathepsin L1	24	18	35.8	28.1	12.0	72.2
Thimet oligopeptidase	2	4	3.0	6.3	35.4	**−**70.7
MMP25	4	2	6.0	3.1	31.3	62.6
Thrombin	4	2	6.0	3.1	31.3	62.6
Signal peptidase complex catalytic subunit	8	11	11.9	17.2	18.0	**−**54.0
MMP14	30	35	44.8	54.7	10.0	**−**49.8
Kallikrein 2	5	3	7.5	4.7	22.8	45.7
MMP12	63	69	94.0	107.8	6.8	**−**41.0
MMP1	26	30	38.8	46.9	9.4	**−**37.7
Cathepsin S	15	12	22.4	18.8	8.8	26.5
Triptidyl-peptidase 1	3	4	4.5	6.3	16.5	−16.5
ADAMTS5	18	20	26.9	31.3	7.5	−15.1
Kallikrein 5	4	3	6.0	4.7	12.0	12.0
MMP2	28	30	41.8	46.9	5.7	−11.5
Cathepsin K	16	14	23.9	21.9	4.4	8.8
MMP7	42	43	62.7	67.2	3.5	−3.5
Meprin A	38	36	56.7	56.3	0.4	0.8
Calpain 2	10	10	14.9	15.6	2.3	0.0
Neprilysin	10	10	14.9	15.6	2.3	0.0

1 Frequency distribution analysis based on all peptides (n = 131).

Mathematical calculations are based on the following parameter and calculations:

**occ(up)** = Sum of all occurrences for each individual protease in the up-regulated peptides,

**occ(down)** = Sum of all occurrences for each individual protease in the down-regulated peptides,

**N(up)** = Total number of peptides being up-regulated,

**N(down)** = Total number of peptides being down-regulated,

**% frequency(up)** = (occ(up)/N(up)) * 100.

**% frequency(down)** = (occ(down)/N(down)) * 100.

**% frequency difference ratio** = | ((freq%(up) − freq%(down))/(freq%(up)+freq%(down)) * 100 |.

**Frequency scores** = %freq * (occ(up)-occ(down)).

## Discussion

Based on the comparison of 33 newly diagnosed patients with RA and 30 healthy controls, we were able to identify 292 potential urinary biomarkers associated with the diagnosis of RA. Of these, 70 were sequenced and identified by MS/MS, and 39 used to develop a biomarker model for RA after adjusting for age and gender. This is the first study to show that a urinary biomarker model has potential to assist with the early diagnosis of RA, with good sensitivity and specificity (88% and 93% respectively). Although repeat analysis with better matched controls is required to evaluate if the model is truly specific for RA, these initial results compare favourably to ACPA [6]. Furthermore, a panel of biomarkers like this usually performs better as a diagnostic tool than single or few biomarkers alone [29].

Urinary peptides and protein fragments are the end products of upstream proteolytic processes so the differential urinary excretion of peptides between controls and RA patients may indicate their role in the pathophysiology of the disease. Some of the protein fragments identified originated from proteins known to be up/down regulated in RA, including collagens [30], [31], gelsolin [32], and fibrinogen alpha [7], [33]. To identify which proteases might be responsible for the urinary biomarkers identified, we performed a Proteasix prediction analysis. This suggested an increased activity of KLK-6 and PLG and a reduced activity of cathepsin B, MMP3, MMP8, MMP9 and MMP13.

The kallikrein-kinin system with activation plasma (KLKB1) as well as tissue kallikrein (KLK1) has been implicated in inflammation, and raised levels of both have been found in plasma and synovial fluid of patients with RA [34]–[38]. Less is known about the role of KLK6 in the pathogenesis of RA, but KLK6 has been found in synovial fluid in patients with psoriatic arthritis [39], and KLK6 promotes survival of murine lymphocytes with actions on proteinase-activated receptor 1 [40].

PLG has been implicated in early RA and although the mechanism remains to be fully established, it may cleave components of the complement system and activate protease-activated receptors and MMPs [41]. Both MMPs and cathepsin B contribute to joint destruction in RA [42], [43] and elevated levels of serum MMP1 and MMP3 correlate well with the progression of erosive disease in early disease [44]. It is therefore unexpected that the protease prediction analysis consistently suggested down-regulation of MMPs and cathepsin B in our RA cohort. Future studies are required to evaluate and explain these findings which may reflect technical elements of our approach or allude to as yet unexplained renal biology in RA patients.

Further validation is also needed to confirm the diagnostic value of urinary biomarkers in early RA. In particular, comparison should be made between the urinary proteome of patients with RA and aged and gender matched patients with other chronic inflammatory conditions and arthritidies. This will establish if the peptides and peptidases identified are specific to RA or reflect chronic inflammation and joint degradation in general. It would also be informative to take into account disease characteristics such as the 2010 classification score and the presence of erosions. This was not possible in the current study due to the small sample size, which also has implications for the power of the study as a whole. It will therefore be necessary to use a bigger and better characterised cohort when validating our findings. This will allow for estimations of sensitivity and specificity of our biomarker profile for diagnostic purposes and will also allow more powerful correlations between individual peptides and disease markers and characteristics. The current study is based on a cross-sectional design but a longitudinal design would be required to monitor disease progression, to investigate if the urinary proteome at baseline is predictive of outcome. A longitudinal design would also allow monitoring changes in the urinary proteome over time in the same patients with correlation to clinical assessments and response to treatment. In cardiovascular disease, for example, it has been shown that the urinary biomarker pattern becomes healthier in patients who had received treatment [14].

## Supporting Information

Table S1 31 biomarkers showed a correlation with age, gender or both.
(XLS)
